# Our Reading Room

**Published:** 1873-12

**Authors:** 


We shall keep regularly filed at the office of the Journal, No. 20 Line Street, near the Kimball House, all of our *exchanges;* a list of which will appear monthly in the Journal. We cordially invite our subscribers, and other physicians, who may be passing through Atlanta to call at our office and read at their pleasure. A small library of standard medical works, and the new monographs as they appear from the press, will likewise be at the service of visitors.

We keep on file, also, illustrated and priced Catalogues of Instruments, Books, Pharmaceutical and Chemical Preparations, Chemical and Philosophical Apparatus, &c., for the information of our friends; and will aid those who may desire it, in making purchase of such articles as they may need. Many of the wants of physicians in the country can now be most conveniently supplied, through the mails, at their own post-offices.

---

We have received from Messrs. G. & C. Merriam, the publishers, a copy of the large quarto edition of Webster's Dictionary. This work is a magnificent monument to the memory of Noah Webster, and the recognized standard on this Continent. It is as indispensable to the scholar as the pen and ink.

